# Multi-Focus Image Fusion and Depth Map Estimation Based on Iterative Region Splitting Techniques

**DOI:** 10.3390/jimaging5090073

**Published:** 2019-09-02

**Authors:** Wen-Nung Lie, Chia-Che Ho

**Affiliations:** Department of Electrical Engineering, Center for Innovative Research on Aging Society (CIRAS), and Advanced Institute of Manufacturing with High-tech Innovations (AIM-HI), National Chung Cheng University, Chia-Yi 621, Taiwan

**Keywords:** depth from focus, all-in-focus, multi-focus, image fusion, depth image

## Abstract

In this paper, a multi-focus image stack captured by varying positions of the imaging plane is processed to synthesize an all-in-focus (AIF) image and estimate its corresponding depth map. Compared with traditional methods (e.g., pixel- and block-based techniques), our focus-based measures are calculated based on irregularly shaped regions that have been refined or split in an iterative manner, to adapt to different image contents. An initial all-focus image is first computed, which is then segmented to get a region map. Spatial-focal property for each region is then analyzed to determine whether a region should be iteratively split into sub-regions. After iterative splitting, the final region map is used to perform regionally best focusing, based on the Winner-take-all (WTA) strategy, i.e., choosing the best focused pixels from image stack. The depth image can be easily converted from the resulting label image, where the label for each pixel represents the image index from which the pixel with the best focus is chosen. Regions whose focus profiles are not confident in getting a winner of the best focus will resort to spatial propagation from neighboring confident regions. Our experiments show that the adaptive region-splitting algorithm outperforms other state-of-the-art methods or commercial software in synthesis quality (in terms of a well-known Q metric), depth maps (in terms of subjective quality), and processing speed (with a gain of 17.81~40.43%).

## 1. Introduction

3D scanning has been important in industry for several decades. The main methods include passive and active types. The active type usually uses a device to illuminate light (specifically in the near infrared band) and receive it for measuring distances or sense surface variation based on the Time-of-Flight (TOF) [1] or Light-encoding (e.g., Kinect) [1] principles, respectively. The passive type, however, computes three-dimensional surfaces or distance values based on the natural light reflected from objects’ surfaces. The passive method is characterized by low hardware cost, but large computational load. The active method, on the other hand, has a high hardware cost and large measurement noise.

“Depth from Focus” (DFF) [2,3] algorithms were developed for 3D measurement or scanning several years ago. In contrast to traditional stereo vision, they use a monocular camera which varies focus lengths or changes imaging plane positions for the same scene. For example, the focal sweep camera [4] uses a high-speed image sensor translated with respect to the lens (often a duration of 200~500 ms to capture a stack of 24~60 images), or a liquid lens whose focal length is electronically adjusted as a function of time. Also, light field cameras (such as Lytro, CA, USA and Raytrix, Hamburg, Germany) can be used to capture an instantaneous focal stack by trading off the spatial resolution. Since all pictures for DFF are taken at the same viewing direction, the occlusion problem, as in stereo matching, can be avoided. Here we will investigate the DFF techniques to synthesize the All-In-Focus (AIF) image and estimate the corresponding depth map based on the multi-focus image stack [5].

Principally, objects located at different distances will present different focuses on resulting pictures due to limited depth of field of the optical system. By varying the camera settings (e.g., focal length of the lens or position of the imaging plane) for the same scene at the same distance, a focal image stack can be obtained, where each of them presents different in-focus regions. It is possible for DFF techniques to reconstruct or generate an AIF image by extracting pixels of best focus from among the focal stack and record the frame indices accordingly. Since each frame index in the stack corresponds to a focal length or an imaging plane position, the object-to-lens distance *p* can be calculated from the well-known optical imaging geometry: $\frac{1}{p}+\frac{1}{q}=\frac{1}{f}$, where *f* is the focal length and *q* is the distance between the lens and the imaging plane. The depth map can then be simply expressed as the index map that leads to the best focus.

The success of DFF/AIF techniques rely on a reliable focus measure for image patches. The focus measure operator often concerns a transformation of the original image patch to enhance its sharpness. The resulting energy of the transformed patch is then calculated as the focus level estimation. Traditional transforms often estimate the spatially high frequency information in a local window to indicate the focusing level, e.g., Laplacian filtering [6] and the variation [2] approaches. Chen et al. [3], however, apply Gaussian (low-pass) filtering to blur the target image and then compare the blurred result with the original one; the difference can then be used to reveal the focus level of the original image. Image quality measure (IQM) [7] was adopted by calculating the average of gradients for pixels within a window. In [8], the modulation transfer function (MTF) is calculated as a ratio between the image contrast and sharpness to indicate focus metric. In [9], the surface areas of the enclosed region around a same given pixel in different focused input images are computed and compared, as a measure, to distinguish focused and blurred regions. In [10], Li et al. present a Multi-scale Image Analysis (MIA) technique to determine the focusing properties of input image pixels. However, their proposed metric is still misjudged on smooth regions and needs a block-based consistency verification procedure for correction. The above metrics might still result in higher focus measurements for blurred or smooth regions due to noise or image degradation, which will certainly degrade the reconstructed AIF image when the maximum selection rule is adopted.

DFF algorithms can be categorized into pixel-, block-, and region-based [11]. This kind of categorization depends on the area where a focus measure is computed. For pixel-based algorithms [12,13], a pixel in the AIF image is often calculated as a weighted average of the collocated pixels in the original focal stack. However, these kinds of methods will yield a low-quality or noisy AIF image in the presence of noise. Pertuz et al. [13] proposed a selective weighting scheme (a linear combination of selected pixels with higher focus measures) so as to reduce the noise in the AIF image. Other methods include post-optimization [14] on the resulting weight maps before image fusion is performed. For block- [15,16] or patch-based algorithms, a regular shape often results in blocking or ringing artifacts and probably fails near region boundaries. To solve this problem, [11] proposed a region-based algorithm, where the focus measure is calculated for each segmented region of arbitrary shape. The “average image” calculated from the focal stack is incurred segmentation by means of the well-known mean-shift algorithm to define initial regions. In their work, region definitions are however fixed and not further refined. Lee et al. [17] also proposed region-adaptive fusion from focal stack images. A two-level DWT (Digital Wavelets Transform) is first applied to each frame of the focal stack. The focus profile of each pixel is then calculated from the detailed high-frequency sub-bands. All pixels are classified into three kinds of regions (according to the number of peaks in the considered focus profile) and different fusion rules are applied to different kinds of regions. Please note that in their work, pixels classified with the same kind of region in AIF are not extracted from the same image, but only applied with the same fusion strategy. Zhang et al. [18] proposed finding boundaries between the focused and defocused regions, from which the source images could be naturally separated into regions with the same focus conditions. Their method, however, relies on the use of multi-scale morphological gradient operators to improve the precision of boundary detection and focused region detection. Adaptive region segmentation can be also achieved via spatial quadtree decomposition [16,19,20], which is used to define hierarchical regions for focus measures. In contrast to the arbitrary region shapes in [11] and [18], quadtree methods require a metric to determine whether a block will be WTA (Winner-Take-All)-fused or decomposed into four smaller ones. However, they might suffer from over-segmentations into smaller regions, due to regular quad decomposition for a region. Using this method, their works were focused on fusion from two images only and no experimental reports were given for extension to multi-focus image sets whose number of images is larger than 2.

In view of the fusion algorithm, AIF algorithms are categorized into focal-weighting [13], WTA [12,21], and weight-optimized [14]. The focal-weighting method computes a weight of 0.1–1.0 for each image in the stack when synthesizing a specific pixel or block of given coordinate along the focal axis, i.e., weights are focal-position-dependent. The weight-optimized algorithms, however, refine the pixel-dependent weights subject to certain smoothness conditions. Though they are capable of getting better AIF and depth estimation, it seems time consuming and unsuitable for real-time applications. On the other hand, WTA seems to be a special case of the focal-weighting strategy that only one focal position in the stack is selected and has a weight of 1.0, while others have zero weights. WTA is simpler and popular in many applications. There are also some modified algorithms to improve the WTA scheme. For example, [21] proposed using gradually changing sizes of smoothing kernels for eliminating visual artifacts in boundary regions of the initially WTA-fused AIF image. Liu et al. [22], on the other hand, proposed a CNN model for simultaneous activity level measure (feature extraction) and fusion rule design (classification). This deep learning approach, though new, is not appropriate for generating the depths of the scenario. Xiao et al. [23] first extracted image depth information through the inhomogeneous diffusion equation for simulating the optical imaging system, classified pixels into three types of regions (clear, fuzzy, and transition) according to depth information, and finally generated the fused image based on the clear and transition pixels. Their method actually belongs to a kind of DFD (Depth from Defocus, in contrast to DFF herein), which often suffers from inaccurate depth estimation from limited number (often 1 or 2) of defocused images. Some methods [24,25,26] tried to construct global focus detection algorithms, making them get free of block artifacts and reducing the loss of contrast in the fused image. For example, references [24,26] proposed to decompose each of the multi-focus source images into cartoon and texture content; the two different contents are fused respectively and then combined to obtain the all-in-focus image. Unfortunately, only fusion results for two source images were reported. In [26], the authors also applied their cartoon/texture decomposition and sparse representation algorithm for multi-modality (such as medical PET/MRI, or infrared/visible) image fusion.

**Figure 1:**
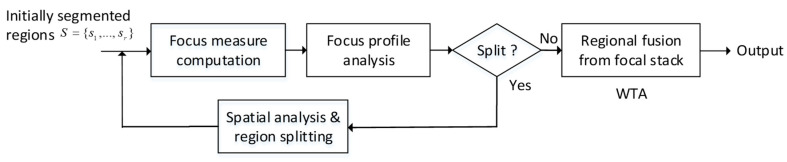
Our region-splitting algorithm for multi-focus image fusion.

Though many AIF algorithms have been proposed up to now, a large part of them are targeted at a stack of two images only and thus unsuitable for extension to depth estimation for larger image stacks (often up to several dozen) in industrial applications. As introduced, region-based methods play tradeoffs between pixel- and block-based algorithms in aspects of complexity and quality. However, a content-adaptive region determination algorithm is seldom developed. We are then motivated by the above two situations. In this paper, we extend our prior work [1] to propose a region- (in spatial domain) and WTA-based (in focal domain) algorithm which overcomes the above two problems and use industrially captured image dataset for testing. Figure 1 illustrates our iterative region-splitting algorithm for AIF image fusion and depth estimation. Differing from traditional region-based algorithms (e.g. [11,16,18,19,20]), our definition of focusing regions is subject to iterative focal-spatial analysis (rather than spatial analysis only, as in [11,18]). No limitation on the manner of region splitting (unlike quadtree [16,19,20]) also makes our algorithm less affected by the possible blocking effect.

First of all, the image domain is initially segmented into regions based on an “initial AIF image” synthesized based on a simple pixel-wise and focal-weighting scheme (Section 2). The focus measures are then computed for each region definition at different focal positions. By analyzing the focus profile (a curve of regional focus measure along the focal axis) for each region, we are able to determine if the targeted region should be split. If the focus profile meets the no-splitting criterion, WTA-fusion along the focal axis is performed to get the fused result for the region. Otherwise, the region is split into subparts after spatial analysis and each divided part is incurred a recursive process for focus computation and analysis. The depth map can be obtained from the AIF image by assigning, to each region, the index of the frame that has the best focus measure (each index corresponds to a focal position and object distance). To sum up, we propose a region-based (in spatial domain) and WTA-based (in focal domain) algorithm for DFF.

## 2. Initial AIF Image Computation and Region Segmentation

First, we define a focal stack $I=\left\{I^{1},\ldots I^{k},\ldots ,I^{K}\right\}$ (where *k* is the image index corresponding to an imaging-plane position and *K* is the number of images contained in the stack). Our aim is to synthesize an AIF image *P* and estimate the depth map *D* from **I**. It is known that the high frequency strength around a pixel can be used as a metric of focusing. High-focusing pixels will be given larger weights in image fusion from the focal stack. The following formula [14] is used here:

(1) $$y_{i}^{k}=\theta_{i}^{k}{\left[\mathrm{erf}\left(\frac{\left|g_{i}^{k}\right|}{\sigma^{k}}\right)\right]}^{K}$$

where subscript *i* represents the pixel index, the superscript *k* is the image index, $g_{i}^{k}$ stands for the gradient, $\sigma^{k}$ indicates the variance of gradients for the *k*-th image, erf(.) stands for Gaussian error function, $\theta_{i}^{k}$ represents the frequency of non-zero gradients around pixel *i* in the *k*-th image, $y_{i}^{k}$ is the weight at pixel *i* of the *k*-th image, and *K* here is an exponent. Therefore, the *k*-th image in the stack has its corresponding pixel-weighting map. The weighting map stack can then be adopted to synthesize an initial AIF image *P* as:

(2) $$P_{i}=\sum_{k=1}^{K}w_{i}^{k}I_{i}^{k}$$

(3) $$w_{i}^{k}=\frac{y_{i}^{k}}{Y_{i}},\;\;\;\;Y_{i}=\overset{K}{\underset{k=1}{\sum }}y_{i}^{k},$$

where $I_{i}^{k}$ stands for the intensity of pixel *i* in the *k*-th image, and *P_i_* is the value of pixel *i* in *P*. Obviously, all $w_{i}^{k}$’s are summed to 1.0 for any given pixel *i*.

Region segmentation technique, e.g., the mean-shift segmentation algorithm [27] in OpenCv, is applied to the initial AIF image *P* to get an initial region set $S=\left\{s_{1},\ldots ,s_{r}\right\}$, where *r* is the number of segmented regions, which depends on some parameters (e.g., “spatialRadius” and “colorRadius”) to control the average of color and space together to form a segmentation. Compared to the “average image” derived in [11], i.e.,

(4) $$P_{i}=\frac{1}{K}\sum_{k=1}^{K}I_{i}^{k},$$

our initial AIF image *P* (Equation (2)) is capable of achieving more focusing, getting more accurate segmentation, and then better/faster convergence in region splitting (see experimental section).

## 3. Spatial-Focal Analysis for Iterative Region Splitting

### 3.1. Focus Measure

According to Figure 1, the initial region set *S* should be subject to focus profile analysis and further refinement (splitting). Here, the variation function in [2] is adopted as our regional focus measure:

(5) $$F_{s}^{k}=\frac{1}{\left|S\right|}\overset{}{\underset{\left(x,y\right)\in s}{\sum }}{\left[I_{Laplacian}^{k}\left(x,y\right)-\mu_{s}^{k}\right]}^{2},$$

(6) $$\mu_{s}^{k}=\frac{1}{\left|s\right|}\sum_{\left(x,y\right)\in s}^{}I_{Laplacian}^{k}(x,y),$$

where (*x*,*y*) stands for image coordinates, *s* is a given region, |s| represents the size of *s*, $I_{Laplacian}^{k}$ is the Laplacian response for the *k*-th image, $\mu_{s}^{k}$ represents the average of $I_{Laplacian}^{k}$ for the region *s*, and $F_{s}^{k}$ is the focus measure for region *s* in the *k*-th image.

According to our experience from experiments, a universal measure that is capable of distinguishing focusing/defocusing for every kind of regions (especially, plain or textureless regions) is hard to find. Thus, the traditional measure of variation is adopted here and more emphases will be placed on spatial-focal analysis that follows.

### 3.2. Spatial-Focal Analysis

**Figure 2:**
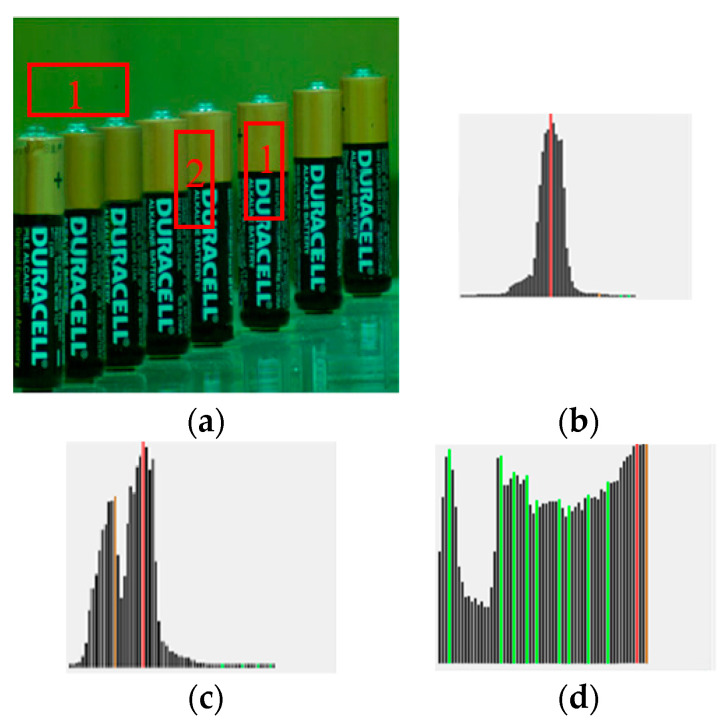
The shape of a focus profile $FP_{s}$, (**a**) Regions 1,2, and 3 for analysis, (**b**) single peak corresponding to region 1, (**c**) two-peak corresponding to region 2, (**d**) multi-peak corresponding to region 3.

To decide whether region *s* should be split, a focus profile analysis in focal axis is conducted. The focus profile of a region *s* is defined to be the curve of focus measures at different focal positions *k*, i.e., $FP_{s}^{}=\left\{F_{s}^{1},\ldots ,F_{s}^{k},\ldots ,F_{s}^{K}\right\}$. Essentially, a single outstanding peak (at position $k^{*}$) *FP_s_* in (e.g., Figure 2b) represents a very good focusing at image k^*^ and will make WTA a confident success. Pixels in region *s* of image k^*^ will be selected for rendering in AIF image, i.e.,

(7) $$k^{*}=\arg\;\underset{k}{\max}\left(F_{s_{h\left(i\right)}}^{k}\right)$$

(8) $$P_{i}=I_{i}^{k^{*}}$$

where $s_{h\left(i\right)}$ represents the region where pixel *i* is located. Contrarily, for bi-modal behavior (e.g., Figure 2c), or even worse (e.g., multi-modal (Figure 2d), flat, or random), the WTA strategy might fail due to multiple objects of different depths in *s*. Therefore, the region *s* should be subject to further splitting so that each sub-region contains only objects of a depth and the corresponding focus profile presents a good single peak.

**Figure 3:**
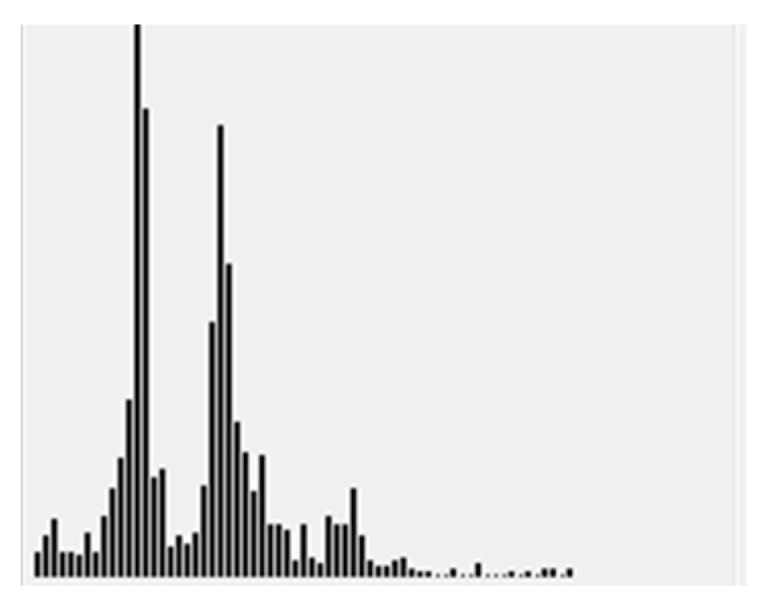
An example of label histogram $LH_{s}$.

For spatial-domain analysis, a label histogram (LH) is generated for region *s*. Denote $LH_{s}$ as the histogram about the image number of best focusing (i.e., the winner) for each pixel in region *s*. The regional focus measures in Equations (5) and (6) are changed for pixels as:

(9) $$F_{\left(x,y\right)}^{k}={\left(I_{Laplacian}^{k}\left(x,y\right)-\mu_{\left(x,y\right)}^{k}\right)}^{2},$$

(10) $$\mu_{\left(x,y\right)}^{k}=\frac{1}{\left|X\right|}\overset{}{\underset{\left(x,y\right)\in X}{\sum }}I_{Laplacian}^{k}\left(x,y\right),$$

where *X* represents a window centered at (*x*,*y*). The analysis of $LH_{s}$, as shown in Figure 3, is similar to $FP_{s}$ in Figure 2. If there is a two- or multi-modes in $LH_{s}$, a splitting into sub-regions is required.

**Figure 4:**
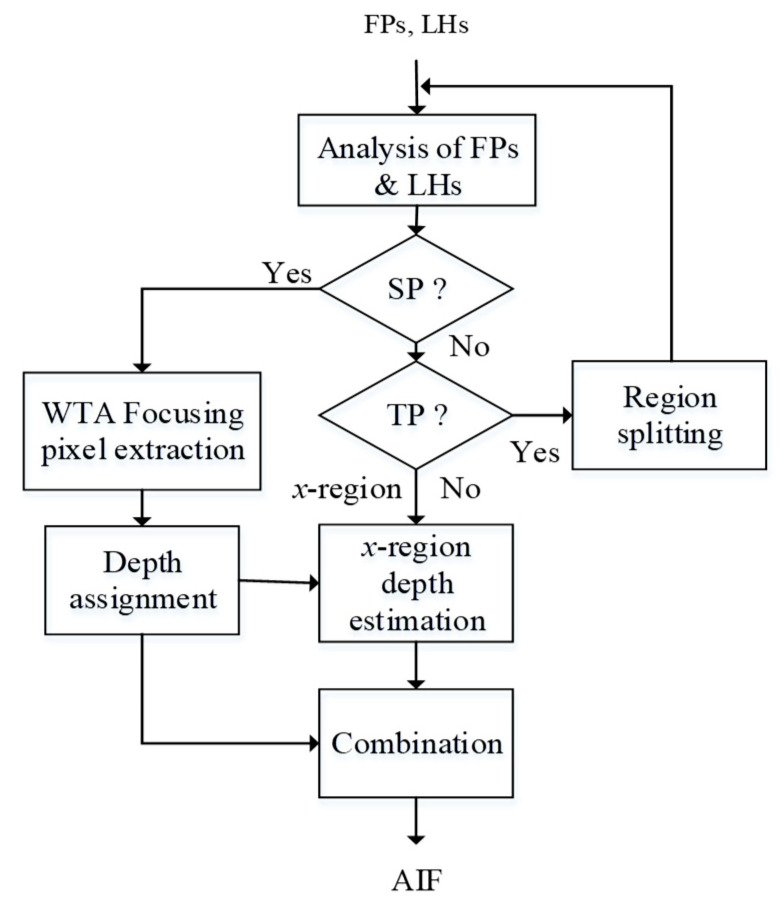
Flow chart of the spatial-focal analysis.

Since $LH_{s}$ and $FP_{s}$ represent feature descriptions along the spatial and focal domain, respectively, the combination of these two features is capable of providing more information for region splitting. Joint analysis of $FP_{s}$ and $LH_{s}$ can be described in Figure 4. $FP_{s}$ and $LH_{s}$ are first analyzed to see whether they satisfy some SP (single-peak) criteria. If they do, WTA can be applied to extract the focusing pixels and assign the corresponding depth value. Otherwise, it turns to the analysis of spatial behavior $LH_{s}$, where the Otsu’s thresholding algorithm [28] is applied to see whether it belongs to the TP (two-peak) category. For TP-type regions, they need to be split into sub-regions, or classified as *x*-regions, otherwise. For *x*-regions, their processing for focusing pixel extraction and depth estimation should rely on support information propagated from neighboring regions. After each region is classified and all *x*-regions are processed, the final AIF can be obtained.

Please note that our SP test is based on both focal and spatial analyses over a region, while the TP test is simply based on spatial analysis of the same region. WTA fusion is applied to a region only wherein the stricter SP criteria are met. If they are not met, a TP criterion in spatial domain is tested for splitting into sub-regions. Usually, the TP criterion is looser so that regions unsatisfying SP criteria are easier to be further split based on their spatial feature and only a limited number of *x*-regions are classified.

**Figure 5:**
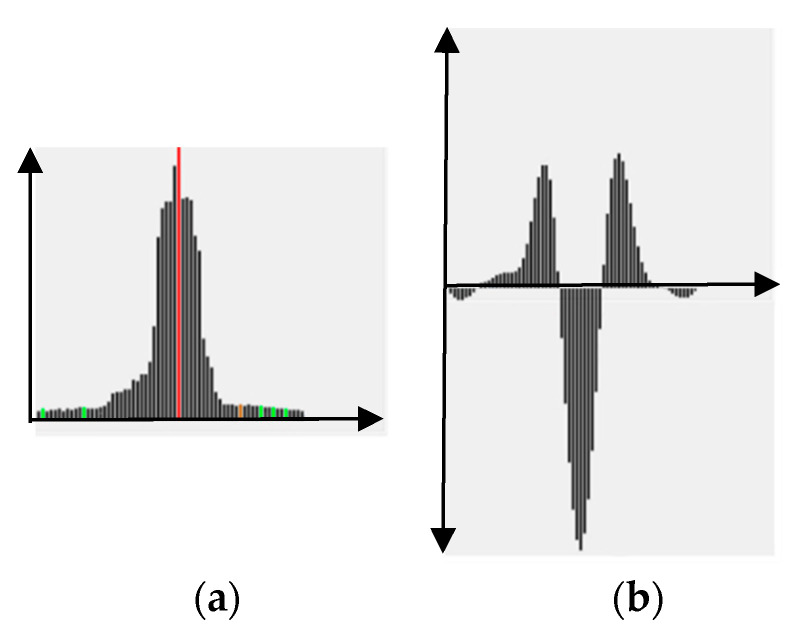
The curves of (**a**) $FP_{s}$ and (**b**) its second derivative.

**Figure 6:**
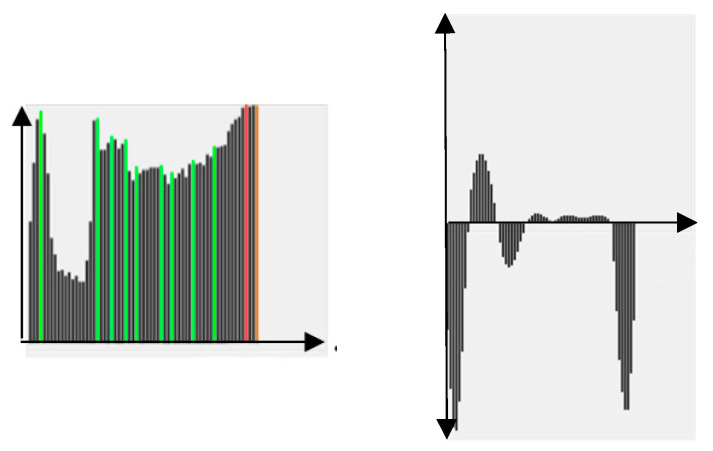
The $FP_{s}$ curve that is not SP-type, but satisfies the ZC and area conditions.

**Figure 7:**
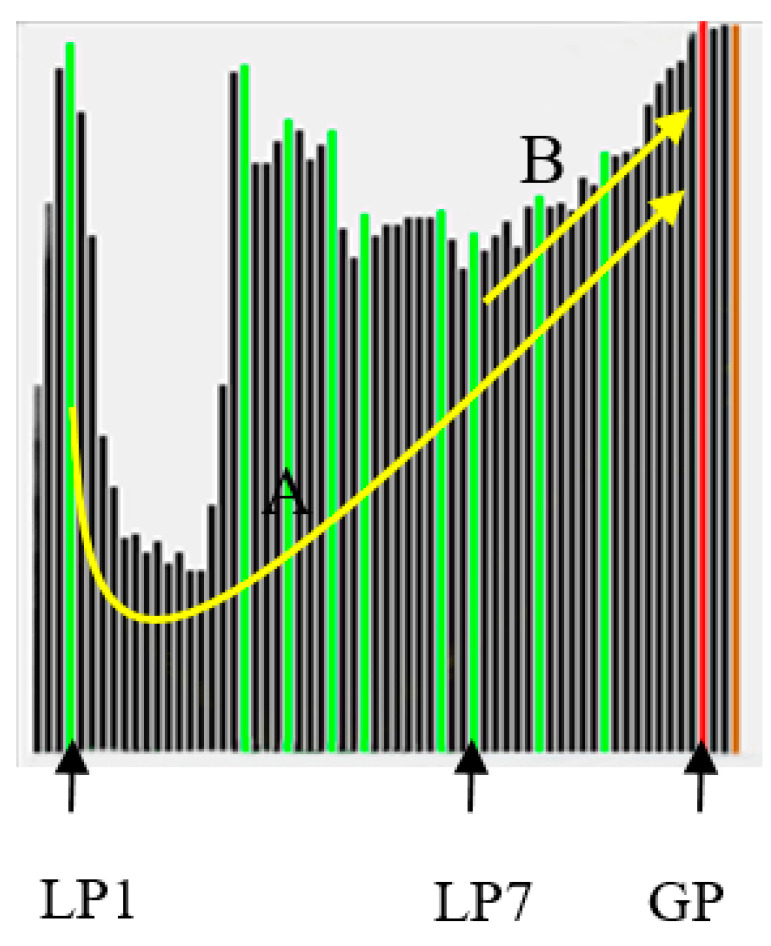
The measure of curve convexity/concavity for each peak $LP_{j}$, with respect to the global peak *GP*.

According to some experimental observationhs (Figure 5, Figure 6 and Figure 7), we propose three SP tests on $FP_{s}$ and $LH_{s}$ as follows.

(T1) The first test is to calculate the second derivative of $FP_{s}$ and count the number of zero-crossings (ZC). For SP curves, the ZC is normally 4 and the positive and the negative parts have similar areas, as shown in Figure 5. If the above conditions are satisfied, then set an indicator $SM_{1}$ to “True”, otherwise, to “False”. Please note that this is only a necessary condition since we can find non-SP curves also satisfying T1. See, for example, Figure 6, where the $FP_{s}$ curve is obviously not SP-type, but satisfies the ZC and area conditions.

(T2) A second indicator $SM_{2}$, built on the analysis of local peaks of $FP_{s}$, is calculated. Local peaks of *FP_s_* are determined, then a convexity/concavity indicator γ for each *j*-th peak ($LP_{j}$), with respect to the global peak (*GP*), is calculated as:

(11) $$\gamma_{LP_{j}}^{}=\frac{1}{\left|GP-LP_{j}+1\right|}\sum_{i=LP_{j}}^{GP}U\left(F_{s}^{i}-F_{s}^{LP_{j}}-thd\right),$$

where $LP_{j}$ and *GP* represent the corresponding image indices of the local and global peaks, *thd* represents a threshold, and *U*(.) is the mathematic step function (i.e., *U*(*x*) = 1 for *x* > 0 and *U*(*x*) = 0 for *x* ≤ 0). We take the curve in Figure 7 as an example, where $LP_{1}$ and $LP_{7}$ are two local-peak positions and *GP* is the global-peak position. Since the curve A from $LP_{1}$ to *GP* is decreasing and then increasing, $\gamma_{LP_{1}}$ is small, while that for $\gamma_{LP_{7}}$ (curve B, increasing) is large. A larger $\gamma_{LP_{j}}$ represents high convexity probably existing in an SP curve. Please note that our computation for $\gamma_{LP_{j}}$ is robust to noise in the curve.

By combining $\gamma_{LP_{j}}$ from each local peak $LP_{j}$, $SM_{2}$ is expressed as:

(12) $$SM_{2}=\frac{1}{H}\sum_{j}\left(\gamma_{LP_{j}}\cdot \beta_{LP_{j}}\cdot {\tilde{F}}_{s}^{LP_{j}}\right),$$

where

(13) $$\beta_{LP_{j}}=\left|LP_{j}-GP\right|,$$

(14) $$H=\sum_{j}\left(\gamma_{LP_{j}}\cdot \beta_{LP_{j}}\right).$$

and ${\tilde{F}}_{s}^{LP_{j}}$ is a normalized (0~1.0) version of $F_{s}^{LP_{j}}$ (i.e., set ${\tilde{F}}_{s}^{GP}$ = 1.0). It can be seen from Equation (12) that the contribution of ${\tilde{F}}_{s}^{LP_{j}}$ from each local peak $LP_{j}$ is distance-weighted by β. If $LP_{j}$ is near *GP*, it could be a noise and given a small weight. $SM_{2}$ is capable of measuring the curve trend from all local peaks to the global peak. A larger $SM_{2}$ (e.g., approximate to 1.0) indicates a higher likelihood of being an SP curve.

(T3) The 3rd test is to apply Equations (11)–(14) to the curve $LH_{s}$ and name the corresponding indicator as $SM_{3}$.

For each region *s*, *SP* is identified when *T1* ~ *T3* are all satisfied. Otherwise, *T4* as follows will be tested.

(T4) The 4th indicator *SM*_4_ is to verify the bi-modality (two-peak, *TP*) of *LH_S_*. First, traditional Otsu’s thresholding algorithm [28] is adopted to binarize *LH_S_* and produce two clusters (their probabilities are denoted as $P_{0}$ and $P_{1},\;P_{0}+P_{1}=1.0$. If both $P_{0}$ and $P_{1}$ are between 0.4~0.6 (i.e., sizes of the two clusters are approximate) and the frame distance between these two peaks is greater than a threshold, a *TP* characteristic of $LH_{s}$ is then identified and $SM_{4}$ is set to “*True*”; otherwise, “*False*”.

**Figure 8:**
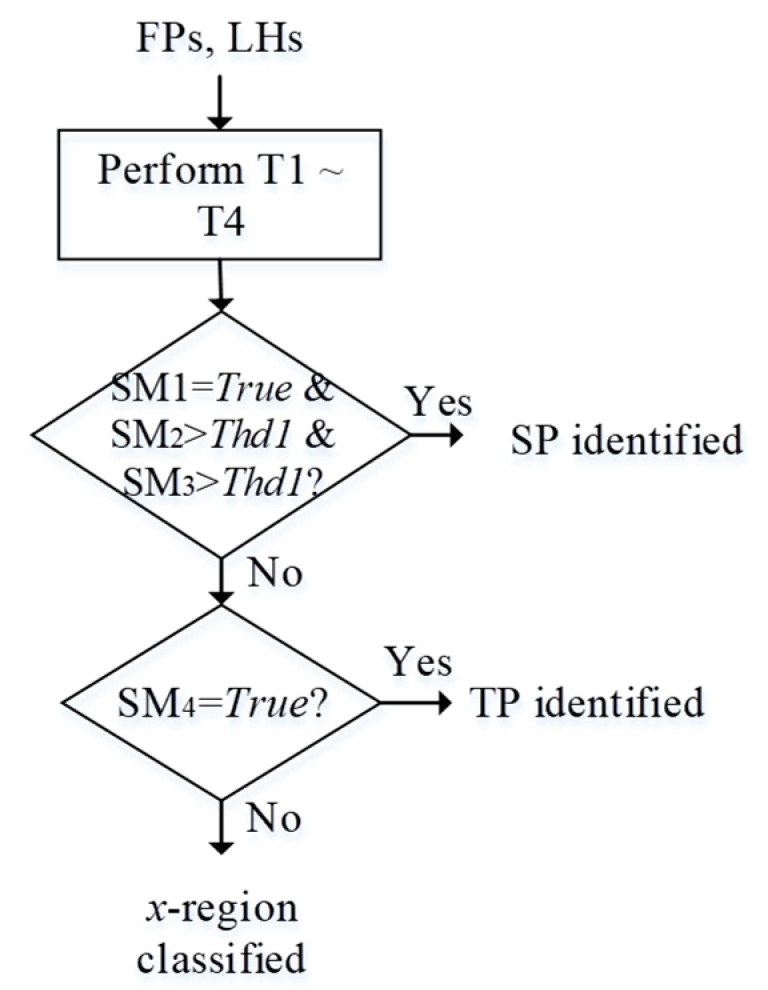
Test flow for the classification of a region *s* into *SP*, *TP*, or *x*-region.

If T4 is satisfied, a *TP* region is identified. Otherwise, *s* is identified as an *x*-region. The test flow is summarized as in Figure 8, where $Thd_{1}$ is an empirical threshold on both $SM_{2}$ and $SM_{3}$.

### 3.3. Iterative Region Splitting

If a region *s* satisfies T4 (or, identified as TP), it will be split into sub-regions. Our method relies on Otsu’s thresholding in T4, i.e., a pixel is classified into cluster 1 or 2, depending on whether its winner (image) index is larger (cluster 2) or smaller (cluster 1) than the estimated threshold. Notice that it is not guaranteed that pixels in cluster 1 or 2 are connected; i.e., either the region of cluster 1 or cluster 2 may not be connected after splitting. Each connected sub-region of *s* will be led to restart of the procesing flow in Figure 8.

### 3.4. Processing of x-Regions

Best focus or depth for regions marked as *x*-type (for examples, whose focus profiles have flat or multi-peak features) cannot be estimated reliably from WTA strategy based on their own pixel data. They can be instead derived from those of neighboring regions successfully classified to SP-type. Though the algorithm of depth propagation [29] can be used to estimate unknown depths of a pixel/region from other adjacent ones, the blending expression in [29] often results in a weighted average which does not necessarily reveal the true depth value. Instead, we adopt a strategy that an *x*-type region will be assigned with a depth value the same as that of the dominant one that is adjacent and has the largest borderline length. Since two *x*-type regions may be adjacent, the one has a larger proportion of known boundary depths will have a higher priority for depth assignment. Once the priority one is assigned with a depth value, it can then be propagated to those adjacent *x*-regions of lower priorities. This depth assignment process is iteratively performed until depths of all *x*-types regions are estimated.

Please note that once the depths (image indices) of the *x*-regions are determined, pixels of best focus can be extracted accordingly.

## 4. Depth Image Post-Processing

Even when we designed three strict tests (T1~T3) to identify SP-type regions, it is still possible that pixels extracted from the peak frame $k^{*}$ do not show better focusing than others. This may result in noise in depth estimates. They need to be further corrected.

First, candidates of depth-noisy regions, denoted as $s_{nz}$, are identified. They are identified to be regions with large depth change (above an empirical threshold $Thd_{2}$) regarding to neighboring regions along ρ% of the outer boundaries. Depth correction here is similar to depth assignment of *x*-regions, i.e., depth correction/replacement of $s_{nz}$ is conducted by that of the dominant neighboring region. This correction is iteratively performed until no regions $s_{nz}$ can be identified.

Our proposed Algorithm 1 is summarized as follows.

**Algorithm 1:** Iterative region splitting for multi-focus image fusion and depth map estimation1 Input a focal stack image set $I=\left\{I^{1},\ldots I^{k},\ldots ,I^{K}\right\}$.2 Form an initial AIF image *P* based on Equations (1)–(3). 3 Initialize region segmentation for *P* by mean-shift algorithm to get an initial region set $S=\left\{s_{1},\ldots ,s_{r}\right\}$. 4 Determine, for each region s, whether it should be split based on spatial-focal analysis in steps 5~8. 5 Compute the focus profile $FP_{s}^{}=\left\{F_{s}^{1},\ldots ,F_{s}^{k},\ldots ,F_{s}^{K}\right\}$ (Equations (5)–(6)). 6 Perform spatial-domain analysis by calculating the label histogram *LH_s_*. 7 Perform joint analysis of *LH_s_* and *FP_s_* based on T1~T4 and classify a regions into SP, TP, or x-region, based on the flow in Figure 8. 8 Iteratively split (based on Otsu algorithm) a region *s* if T4 is satisfied (or, identified as TP) and go to step 4. 9 Perform depth assignments for regions classified as SP and x-regions to form depth map image. 10 Perform depth image post-processing.

## 5. Experiment Results

**Figure 9:**
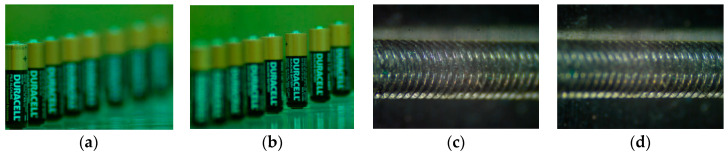

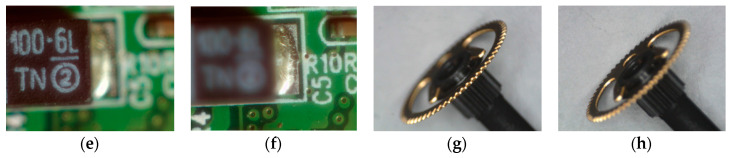
Our test focal stacks (only two images among them are shown). (**a**,**b**) “Battery”, (**c**,**d**) “Screw”, (**e**,**f**) “PCB”, (**g**,**h**) “Tool”.

Four focal stack images for industrial use are captured by adjusting the camera imaging plane at different positions. As shown in Figure 9, the four test image sets include: “Battery” (65 images), “Screw” (48 images), “PCB” (48 images), and “Tool” (7 images), all of 640 × 480 pixels. The parameters are set empirically as: $Thd_{1}$ = 0.8 (Figure 8), $Thd_{2}$ = 0.67 *K, *ρ* = 53 (Section 4), to control final region segmentation and the formation of x-type regions.

**Figure 10:**
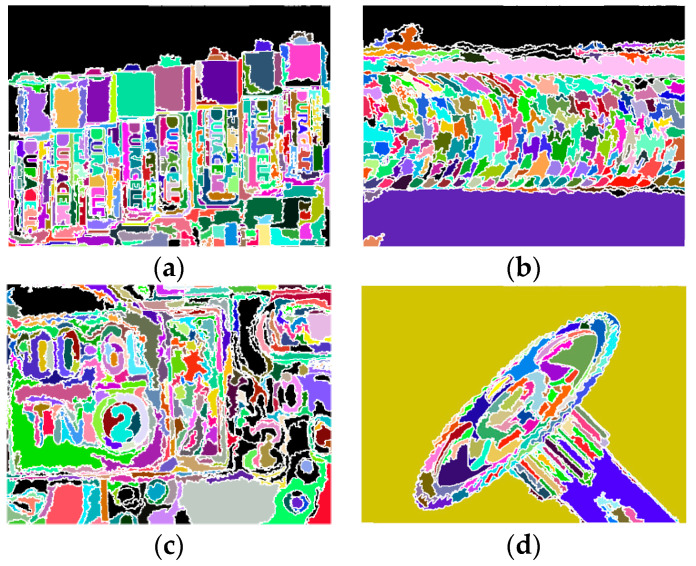
Results of iterative region splitting, *x*-regions are colored in black, (**a**) “Battery”, (**b**) “Screw”, (**c**) “PCB”, (**d**) “Tool”.

Figure 10 shows the region segmentation for the four test images, where *x*-type regions are colored in black. The initial and final numbers of segmented regions are listed below and the numbers of *x*-type regions are also shown in parentheses.

(1) “Battery”: 299 → 367 (*x*-type: 15)
(2) “Screw”: 242 → 256 (*x*-type: 15)
(3) “PCB”: 343 → 367 (*x*-type: 49)
(4) “Tool”: 102 → 102 (*x*-type: 0).

It can be observed that most of the *x*-regions occur at plain backgrounds that have less textures for focus measure, e.g., in top background of “Battery” and “Screw”. This however does not cause any difficulty in identifying SP property for the bottom and whole background of “Screw” and “Tool”, respectively. For “PCB”, the number of regions is increased by 24 after splitting, while 49 out of 367 are classified to be *x*-type. This means at least 49 − 24 = 25 regions in initial segmentation are neither classified as SP nor TP type. According to Figure 10c, *x*-type regions concentrate on the green backgrounds of the right part image. This is similar to the behavior of the green backgrounds in “Battery”. Notable is the result of “Tool”, where no regions are further split and classified as *x*-type. This good behavior also lead to better AIF synthesis and depth map estimation (see the results later).

**Figure 11:**
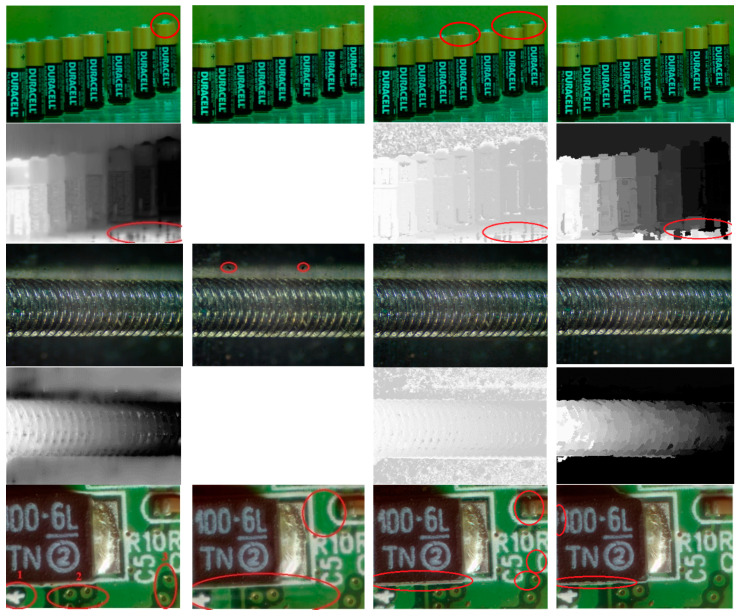

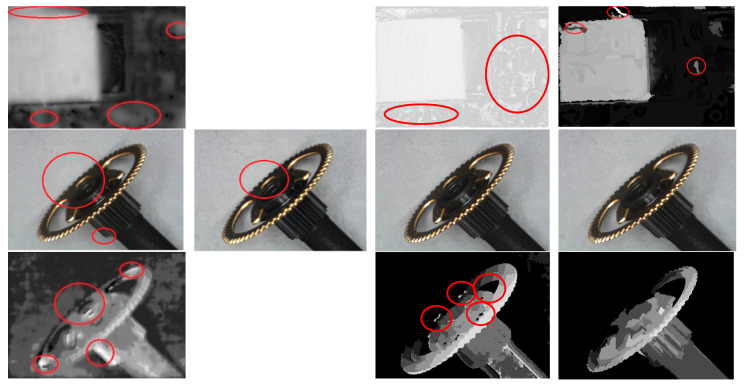
Results of AIF images (**odd rows**) and depth maps (**even rows**) for “Battery”(**rows 1–2**), “Screw” (**rows 3–4**), “PCB” (**rows 5–6**), and “Tool” (**rows 7–8**) obtained by Helicon (**column 1**), Zerene Stacker (**column 2**), [12] (**column 3**), and proposed method (**column 4**).

Figure 11. compares four methods on AIF synthesis and depth map estimation: (1) Helicon, (2) Zerene Stacker, (3) [12], and (4) our proposed method. For visual clarity, all depth maps are scaled to gray levels in 0~255). The Helicon, being a popular commercial software, adopts a pixel-weighting strategy, i.e., it calculates a weight for each pixel according to the image content and then gets final fused result by weighting co-located pixels from all source images. The Zerene Stacker, a commercial software much expensive than Helicon, is featured of accurate and robust alignment, scale correction by interpolation, and advanced stacking algorithm. It can also generate stereo and 3-D rocking animation from a single stack. Reference [12] is a pixel- and WTA-based algorithm, enhanced with post-processing on the resulting AIF image. It also applies point diffusion function to the filtered AIF to generate the re-focused image and depth map, whose number of levels is actually larger than the original image number *K*. Since Zerene Stacker cannot provide the resulting depth maps for the trial version, they are not shown in Figure 11. Depth maps estimated by [12] differs from Helicon’s and ours because of its recalculation by using point-diffusion function.

It is observed from Figure 11 that both our proposed algorithm and Zerene Stacker are capable of achieving a better synthesis quality near electrodes and bodies of the “Battery”, but it seems that Zerene Stacker has better performance on table’s surface. For “Screw”, Zerene Stacker’s result presents two dark dots, while Helicon wrongly estimates the depths for background area and blurs the top boundaries of the screw. For “PCB”, Helicon presents geometrical distortions near the left-top portion of the image, defocusing at “1”, and distorted bright spots at “2” and “3”; Zerene Stacker shows defocusing and redundant textures as indicated in the red circles; [12] gives ripples around object boundaries. For depth estimation, our algorithm has some errors, while Helicon blurs depth boundaries near objects. For “Tool”, our proposed algorithm and [12] show the best results, while Helicon and Zerene Stacker lead to light defocusing. In view of the depth map, Helicon and [12] show several errors. In Figure 11, depth maps for [12] are generally noisy (especially in background areas of the “Battery” and “Screw” and the right part of the “PCB”) and might have a copy-pattern from the color part.

**Figure 12:**
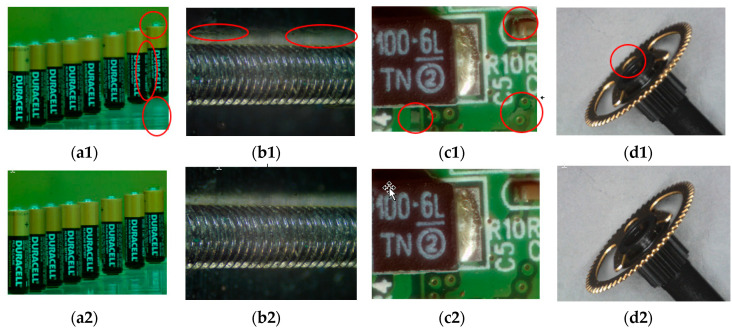
Comparison of results by using Equation (4) (**a1**–**d1**) and Equation (2) proposed (**a2**–**d2**).

Figure 12 shows the results that the initial AIF image *P* calculated from Equation (2) is replaced with the average image calculated by Equation (4) [11], which is then used for the same procedures of initial region segmentation and iterative region splitting. Focusing improvement by Equation (2) against Equation (4) as initial AIF image generation can be found by comparing the parts in red circles. Obviously, Equation (2) is capable of providing better AIF results compared with Equation (4).

To evaluate the performance for AIF fusion in an objective manner, the metric proposed in [30] (*Q*, whose values are between 0.0 and 1.0, and a larger value means better quality) is measured with four methods for comparison. Table 1 illustrates the result, where “*” represents the winner. It is obvious that our proposed algorithm outperforms others except the “Screw” set. This could be due to some spots that are present in the bottom background. However, Figure 11 reveal our superiority in depth estimation compared with Helicon for the top and bottom backgrounds of “Screw”.

Table 2 compares the computing time. The computing platform is based on the Intel Core i7-3770 3.40 GHz with 4 GB RAM for Helicon, Zerene Stacker, and our proposed method. The execution time for [12] is, however, based on a CPU of 2.8 GHz and 16 GB RAM. It is observed that our algorithm (with non-optimized code) is faster than Helicon and Zerene Stacker for nearly all four image sets (with an average gain (defined as (compared_time-our_time)/compared_time) of 17.81%, and 40.43%, respectively), while slightly slower than [12] (−15.54%), especially for small image sets (like “Tool”).

## 6. Conclusions

A region-based algorithm is proposed in this paper to synthesize an All-in-focus image and estimate the corresponding depth image for multi-focus image set. Our contributions come from the following aspects: (1) focus measures are calculated based on irregularly shaped regions so as to adapt to varying image contents (unlike the complex pixel-based and the simple block-based methods); (2) our method is capable of refining or splitting the segmented regions iteratively by analyzing spatial-focal behaviors about the regional focus measure; (3) suitable for both AIF and depth estimation purposes.

Our software simulations show that the proposed region-based method works well in the aspects of fusion quality, depth estimation, and speed, with respect to some commercial software that adopt pixel-based or pixel-weighting strategy. Possible future studies may include further detailed classification of a region *s* (currently, SP, TP, and *x*-type) so as to adopt more suitable arrangements. Other fusion strategies that are more effective than the well-known WTA and simpler than the pixel-weighting can be also investigated.

## Figures and Tables

**Table 1:** Quality assessment for image fusion results. The “*” represents the winner for each case of image stack.

	Battery	Screw	PCB	Tool	Average
Resolution × number of images	640 × 480 × 65	640 × 480 × 48	640 × 480 × 48	640 × 480 × 7	
Helicon	0.32721	0.32846 *	0.3015	0.4087	0.3415
Zerene Stacker	0.27272	0.24245	0.30591	0.4027	0.3059
[12]	0.35688	0.26358	0.45498	0.4750 *	0.3876
proposed	0.38451 *	0.27735	0.48273 *	0.4750 *	0.4049 *

**Table 2:** Comparison of computing time (sec).

	Battery	Screw	PCB	Tool
Resolution × number of images	640 × 480 × 65	640 × 480 × 48	640 × 480 × 48	640 × 480 × 7
Helicon	12.47	7.86	9.06	3.21
Zerene Stacker	19.89	24.84	15.48	2.89
[12]	10.89	7.43	7.48	1.29
proposed	12.605	5.949	7.93	2.068
